# The acceptability of alcohol intoxication management services to users: A mixed methods study

**DOI:** 10.1111/dar.13002

**Published:** 2019-11-26

**Authors:** Andy Irving, Penny Buykx, Yvette Amos, Steve Goodacre, Simon C. Moore, Alicia O'Cathain

**Affiliations:** ^1^ School of Health and Related Research University of Sheffield Sheffield UK; ^2^ School of Humanities and Social Science University of Newcastle Newcastle Australia; ^3^ Violence Research Group, School of Dentistry, Heath Park Cardiff University Cardiff UK; ^4^ Crime and Security Research Institute Cardiff University Cardiff UK

**Keywords:** acceptability, treatment, alcohol, night‐time economy, emergency services

## Abstract

**Introduction and Aims:**

Alcohol Intoxication Management Services (AIMS) provide basic care for intoxication and minor injuries, have been increasingly implemented in urban areas characterised by a large number of premises licensed for the sale and on‐site consumption of alcohol, with the goal of reducing alcohol's burden on emergency services, including referrals into hospital emergency departments. The acceptability of new health services to users is a key effectiveness outcome. The aim was to describe patient experiences when attending an AIMS and document the acceptability of AIMS to users.

**Design and Methods:**

A sequential mixed methods study was undertaken involving semi‐structured interviews with participants from four AIMS followed by a survey of users recruited from six AIMS.

**Results:**

Interviewees (*N* = 19) were positive about the care they received in AIMS and appreciated the friendly, non‐judgemental atmosphere. Survey respondents rated their experience in AIMS positively (on a 0 to 10 Likert scale, mean = 9.34, SD = 1.38, *n* = 188). Frequently given reasons for attendance included drinking alcohol (57%) and minor injury (42%); 24% said they would have attended the emergency department had the AIMS not been available and 6% said they would have preferred to go to the emergency department; 31% indicated they would have felt unsafe without the AIMS.

**Discussion and Conclusions:**

AIMS are acceptable to users. AIMS are likely to address previously unmet demand for a safe space within the night‐time environment.

## Introduction

Alcohol contributes 3.3 million deaths globally and accounts for 5.1% of the global burden of disease 1. Acute alcohol intoxication (AAI) is implicated in antisocial behaviour, accidental and violent injury and sexual assault 2, 3, 4. These harms burden ambulance and police services, and hospital emergency departments (EDs) 5, 6, 7, 8. In parallel to policies of prevention, the need to better manage AAI has been identified as a requirement across a number of jurisdictions in the UK 9 and internationally 10, 11, 12, 13.

Alcohol Intoxication Management Services (AIMS; ‘Drunk Tanks’ in lay terminology) have been proposed for night‐time environments (NTE) to better manage AAI and related outcomes 10. They are typically located in the centre of cities and large towns and operate during periods of high alcohol consumption. AIMS treat those requiring short‐term care for AAI and other minor health problems, but where ED care is not warranted. The purpose is to divert demand away from frontline services and to provide a place of safety for patrons of the NTE who are at risk because of their alcohol consumption, but not those with chronic needs 10.

While AIMS share a common purpose, there is considerable variation in the services provided in terms of facility (e.g. building, ambulance or bus), staffing (e.g. number and mix of volunteers, health‐care professionals) and the presence of security staff 10. There is limited evidence that AIMS are effective in diverting patients away from the emergency care system or provide an acceptable service to users 10, 14, 15. User acceptance is an important factor contributing to effectiveness 16, particularly so for new services that provide alternatives to ED: such as a taxi rather than an ambulance to ED or conveyance to a primary care physician rather than the ED 17. The purpose of this study was to describe patient experiences of AIMS and to assess whether AIMS were acceptable to users.

## Methods

A sequential mixed methods design 18 involved qualitative interviews with users from four AIMS, followed by a survey of users in six AIMS. This work is a component of the EDARA (Evaluating the Diversion of Alcohol‐Related Attendances) study that evaluated the acceptability, effectiveness and cost‐effectiveness of AIMS in providing an alternative to ED attendance for AAI. This study was approved by the National Health Service Research Ethics Committee (REC 3, Health and Care Research Support Centre, Cardiff; Reference: 16/WA/0065; Protocol Number: v4.6 SPON1472‐15; IRAS Project ID: 192273). A Public and Patient Involvement group with experience of alcohol and drug use 19 advised EDARA study design, materials and dissemination, as did policy makers and health‐care practitioners.

### *Participants*

Participants were recruited (from December 2016 to October 2017) from AIMS across England and Wales, UK (see Appendix S1, Supporting Information). AIMS have operating procedures specifying who can attend; typically, those exhibiting uncomplicated intoxication or minor injury. All those attending AIMS were eligible to participate.

#### *Interviewees*

Semi‐structured interviews with 19 AIMS users recruited from four AIMS (Sites A, B, C and D, Appendix S1): seven women and 12 men, aged late teens (*n* = 3), early to mid‐20s (*n* = 14) and over 40 (*n* = 2) years of age. Two AIMS refused permission for this aspect of the research. Forty‐nine AIMS users gave written informed consent to be contacted for interview of whom 20 were reached. Nineteen provided verbal consent for interview by telephone. Three participants were working in the NTE before they attended the AIMS.

#### *Survey respondents*

Based on estimated AIMS attendance numbers, our sample size target was 300 to allow comparison of satisfaction levels between different AIMS models (static and mobile), and to detect a difference of 10% (70% vs. 80%) at ɑ ≤ 0.05 and 80% power. Recruitment occurred during AIMS opening hours and 208 usable surveys were received (Fixed sites: Site A, *n* = 59; Site B, *n* = 39; Site C, *n* = 22; Site G, *n* = 17; Mobile sites: Site F, n = 28; Site H, n = 43, Appendix S1; 53% men, 58% aged 17–24 years, 25% aged 25–34 years and 17% aged 35+ years); 20 were missing information on age and/or gender. An exact response rate cannot be calculated because the number of potential participants approached by AIMS staff is unknown but is estimated to be less than 25%. Respondents were predominantly patrons of the NTE, but free text responses indicated that a small number (*n* < 10) of AIMS users were working in the NTE (e.g. bar staff).

### *Materials*

#### *Interview*

A qualitative topic guide was developed in consultation with the Public and Patient Involvement group and EDARA stakeholders. Topics included the decision to attend the service, what happened while there and at the point of discharge, perceptions of care received and preference for alternative services including the ED. The topic guide was piloted, refined and pilot data were not used in the main analysis.

#### *Survey*

Time constraints meant the survey started while the last interviews were being conducted. The 12‐item questionnaire was developed using from the first nine interviews. Items included reasons for attendance, who they attended with, care received, and ratings of care (eight aspects were assessed using five‐point Likert scales: 1 = ‘very good’ to 5 = ‘very poor’; Appendix S1). An 11‐point satisfaction question (0 = ‘I had a very poor experience’ to 10 = ‘I had a very good experience’) was replicated from the Care Quality Commission ED survey 20.

### *Procedure*

#### *Interviews*

AIMS staff alerted prospective participants to the project as patients neared the end of their stay and were judged sufficiently sober to provide consent. Those interested in participating provided written consent to make contact by telephone within seven days. Up to three contact attempts were made. Interviews commenced by reconfirming participant's identity and consent to be interviewed. Interviews followed the topic guide, were recorded and transcribed verbatim.

#### *Survey*

Respondents either completed the questionnaire while in the AIMS (returned via sealed collection box) or completed it following discharge (returned via pre‐paid post).

### *Analysis*

#### *Interviews*

Interview data were managed using Nvivo 11 software 21. A framework analysis approach 22 was adopted to explore both *a priori* and emergent issues. Analysis was undertaken by AI, PB and AOC. Steps included: (i) reading and rereading of the first three interview transcripts (i.e. familiarisation); (ii) discussion of *a priori* and emerging themes, (iii) development of an initial thematic framework; (iv) reading and coding of all transcripts according to the framework, with some evolution to the framework to account for additional emergent themes arising from parallel interview data collection; (v) development and discussion of a schematic diagram to map the range and nature of the data and to aid further interpretation 23, 24; and (vi) preparation of short Case Reports to illustrate example patient pathways.

#### *Surveys*

Survey data were analysed using SPSS 25. Descriptive statistics characterised responses. Differences by AIMS type were examined using Mann–Whitney tests for scaled responses and χ^2^ tests for binary responses.

## Results

### *Interviews*

Data are presented according to the themes that guided interviews.

#### *Circumstances of AIMS attendance and the decision to attend*

Interview accounts confirm the frequently passive role of AIMS users in their pathway into an AIMS, with some having no recollection of being involved in the decision to attend the AIMS.

‘*I was probably told “we* [i.e. the ambulance staff] think it's a good idea” *so I probably just agreed’* (male, early 20s; PID13).

‘*The decision wasn't made by me, it was made by the*, *I assume*, *a member of staff that came from the centre*’ (male, early 20s; PID6).

‘*They just basically took their wheelchair and brought me to the* [AIMS] *because I think that was the closest medical centre*’ (female, late teens; PID2).

Even in situations where a person decided to attend for themselves, they did not necessarily seek the AIMS out.

‘*I just saw two ambulances outside, and I thought “I'll give it a go*”’ (male, early 20s; PID5).

Case report I exemplifies circumstances surrounding AIMS attendance.

Case report I: A young male was found acutely intoxicated by Street Pastors (a non‐denominational church‐led volunteer group) in an alleyway. He had vomited and was offered water and tissues. The Street Pastors offered treatment at a local AIMS. The patient was picked up by an AIMS affiliated vehicle and escorted by AIMS staff. On arrival, around midnight, the patient was taken into the recovery room with mattresses on the floor. An AIMS volunteer took blood pressure, heart rate, temperature and breathalysed him. He was also given bottles of water and a sick bowl before being allowed to sleep for a while. Three hours later the patient was sufficiently sober to provide a friend's contact details who would accompany him home. AIMS staff called a taxi and discharged him home with his friend. No advice or information was given on alcohol use. When interviewed by researchers he expressed gratitude towards the AIMS staff and was satisfied with the service received. He commented that if the AIMS had not been there it would have been a ‘*hassle*’ for friends to have to look after him. ‘*I'd have probably have been ill in a taxi, so I'd have probably had to pay a lot of money so probably, worse scenario, I'd probably have had to go to the A&E to get sorted out there, but I'm not sure*’ (PID16).

In contrast to patrons of the NTE, AIMS users who were employed in the NTE sometimes knew of their existence and chose to be treated there when injured on the job, as illustrated by a bartender who was assaulted after refusing to serve a customer.

Interviewer: ‘*Did you know about the [AIMS] before?*’

Interviewee: ‘*Thankfully I, yeah, as I mentioned in the club I work in, I'm one of the main first aiders*’ (male, early 20s; PID17).

Case report II exemplifies the pathway into AIMS for someone working in the NTE and provides a sober reflection on the compassionate support offered by AIMS.

Case report II: Shortly after midnight a male bartender, in his early 20s, at a local nightclub refused service to a male customer who then punched the bartender in the face. The bartender was aware of the AIMS from workplace first‐aid training. His manager and attending police officer recommended he attend the AIMS for treatment at the end of his shift. When he arrived, a physical examination of his head was undertaken by a paramedic to check for concussion. His blood pressure and heart rate were recorded, and painkillers and water were offered. The patient was advised to attend the ED for an X‐ray to check whether his cheekbone was broken. He was discharged at 3:00 am and the patient called a friend who collected him by car. Although advised to go directly to the ED, the patient opted to go home and instead attended the ED later that day. At interview, this person indicated he was grateful for the care given, was impressed by the service and was particularly struck by the compassion of staff. ‘*A woman that came in and she was absolutely annihilated from drinking too much and the* [AIMS] *was asking her if she had any way home, a friend to call or a taxi and potentially even the police driving her back to her place so that was pretty cool to see’*. ‘*The compassion, the compassion and the help, the lengths that the council, volunteers and police are willing to go*’ (PID17).

#### *Care and treatment received*

Several interview participants expressed a lack of clarity about what care they received and from whom.

‘*I think it was a nurse, or something, potentially? Or I think the police were there as well? But I'm not too sure what was happening*’ (male, early 20s; PID4).

‘*I can't really remember much about the evening*, […], *but I remember they identified who they were and what they were doing*’ (male, early 20s; PID16).

The premise of AIMS is that many of those exhibiting AAI need only a safe place where they can be observed while they sober up and do not need ED. This function was reflected in some interviews.

‘*They took all my details, name, address, what happened to me, where I was at the time and they gave me water to sober me up. They put an ice pack on my foot, they checked over my foot and chatted me to really*’ (male, early 20s; PID1).

‘*It was basically just a seat and them coming up and chatting and obviously while they're chatting, they're assessing you, aren't they? And just a blanket; that was it*’ (female, mid 20s; PID15).

However, patients rarely received an intervention or advice on their alcohol use, and respondents indicated it may not be feasible to do so due to a lack of capacity.

Interviewer: ‘*Do you think it would be a good idea to offer people some advice or information about alcohol use?*’

Interviewee: ‘*The obvious answer is oh yes, of course, but are they receptive at that point, you know, if they've been taken there? You know for me, in my situation, it would've been a complete waste of time to be honest … I don't know, perhaps for the partners of people who are in there maybe? If they know someone who's got a recurring problem, then there might be advice there, whilst they're waiting for somebody to come round that they could be reading and looking at*’ (female, mid 50s; PID15).

As an alternative to an intervention, one site had an arrangement with a local alcohol service to telephone patients in the following week as a check on their welfare and to offer further support and advice as needed. This follow‐up was acceptable to the one interviewee who reported receiving such a call.

#### *Acceptability of AIMS*

Interviewees were positive about their time in the AIMS. There was a perception of safety coupled with calm, reassuring, care. Even if they were uncertain about who had looked after them and other treatment details. This sense of being in friendly, yet competent, hands appeared to be partly fostered by the style of interaction between staff and patients.

*‘It wasn't like that awkward, you know, atmosphere. There was loads of people there like … joking around with everyone there, even the nurses and it was just nice, I enjoyed it was stupid to say, but I enjoyed being there’* (female, mid 20s; PID9).

*‘It was clean, and everyone was very helpful, quite nice and actually listened to me’* (male, mid 20s; PID12).

The clinical nature of the environment in AIMS also gave reassurance.

*‘Well it just literally reminded me of a hospital which is you know, I expected, as I thought it was just a hospital in a convenient location’* (female, late teens; PID14).

#### *Preferences for place of care*

Some interviewees made reference to AIMS being preferable to the ED, particularly in relation to use of hospital resources. For those preferring AIMS to ED there was acceptance that AAI is an unnecessary burden on frontline health services.

*'It's a lot easier than ferrying people back to A&E which I have no doubt was overstretched as it was Saturday night anyway. Obviously they are adept at dealing with, you know minor, drink related issues which probably the vast majority of those calls are, so obviously freeing up space in A&E’* (male, mid 20s; PID13).

*‘I wouldn't have wanted to go all the way to the hospital to waste anyone's time when it wasn't as serious as it looked’* (male, early 20s; PID11).

#### *What may have happened without AIMS*

The AIMS as a place of safety featured in interview accounts. Case report III describes the experience of a young woman who was separated from her friends and unable to care for herself.

Case report III: A young woman in her early 20s was drinking at home first and then in a nightclub. Having drunk too much alcohol, she went to the toilet where she vomited and fell asleep. Her friends assumed she had gone home and left the nightclub without her. When staff checked the toilets after the club closed around 03:30 am they discovered her slumped in the cubicle. The manager of the club called the AIMS who sent volunteers with a wheelchair to collect her. The young woman felt disoriented at having lost her friends and had no phone signal. On arrival at the AIMS a paramedic, police officer and volunteers provided reassurance, gave the woman water and a sick bowl. They kept her talking and awake, asking her about the circumstances of her night out and her use of alcohol. They also gave her advice on how to stay safe with friends. After approximately 1 hour the patient was encouraged by AIMS staff to contact a friend to pick her up. A taxi was then called by the AIMS staff to collect the patient and her friend. The patient was very grateful to be seen in an AIMS rather than an ED *‘because if you go into A&E as a result of drinking, you're a low priority most of the time, because it's self‐inflicted’*. When interviewed, the patient expressed humiliation about the situation and was very grateful for the AIMS facility and staff taking good care of her. *‘I am really mortified about this; it was the most embarrassing thing that ever happened’*. She suggested that if the AIMS had not been available then she would have been taken out of the nightclub by the staff there and left to find a taxi on her own, which she felt she may not have managed as she was not familiar with the city. *‘Without the* [AIMS] *God knows where I would've ended up’* (PID9).

### *Survey*

Overall, on the 11‐point satisfaction scale, participants rated their experience positively (mean = 9.35, SD = 1.38). A Mann–Whitney test for differences in overall ratings for fixed (mean = 9.28, SD = 1.35, *n* = 124) and mobile (mean = 9.47, SD = 1.46, *n* = 64) AIMS yielded no significant effect (*z* = 1.69, *P* = 0.09). Most survey respondents (67%) rated their overall experience of AIMS positively and at the highest level of 10, a further 30% rated it seven to nine. The Care Quality Commission survey on which this item was based suggests 27% of ED patients rate their experience at 10, 51% rate their experience from seven to nine 26. Moreover, responses to the eight service rating scales (Table 1) indicate AIMS were acceptable to users. Mann–Whitney tests found no differences between fixed and mobile sites.

**Table 1:** Survey descriptive statistics and analysis of service quality scales^a^

	All		Fixed		Mobile			
	*n*	Mean (SD)	*n*	Mean (SD)	*n*	Mean (SD)	*z*	*P*
Service location	190	1.23 (0.52)	124	1.28 (0.58)	66	1.14 (0.39)	1.78	0.07
Safety	194	1.10 (0.42)	128	1.13 (0.48)	66	1.05 (0.27)	1.73	0.08
Comfort and cleanliness	191	1.14 (0.50)	125	1.18 (0.57)	66	1.06 (0.30)	1.80	0.07
Communication	193	1.24 (0.65)	128	1.28 (0.69)	65	1.15 (0.57)	1.47	0.14
Care and compassion	194	1.15 (0.54)	128	1.16 (0.54)	66	1.12 (0.54)	0.89	0.37
Tests and treatment	181	1.32 (0.76)	119	1.32 (0.74)	62	1.32 (0.81)	0.30	0.77
Advice or information	185	1.28 (0.73)	121	1.29 (0.74)	64	1.27 (0.72)	0.29	0.77
How was discharged	154	1.17 (0.53)	100	1.16 (0.44)	54	1.19 (0.68)	0.59	0.55

a Likert scores are from 1 ‘very good’ to 5 ‘very poor’; median was 1 for all quality dimensions and by AIMS type. With Bonferroni correction the threshold for significance is *P* < 0.006.

Referring to Table 2, the most commonly reported reason for AIMS attendance was ‘drinking’, followed by ‘injury’ and ‘feeling unwell’. A small number (*n* = 20) gave other reasons including having lost their friends, feeling vulnerable, wanting help to get home, mental health issues and wanting to use toilet facilities. The majority of those attending AIMS were accompanied by other people. Following arrival, survey respondents reported being looked after by ambulance paramedics, nurses and volunteers, although a small proportion also said they were looked after by the police. Overall, there were no statistically significant differences between mobile and fixed sites, other than those that are directly attributable to differences in service configuration (Appendix S1).

**Table 2:** Descriptive statistics from the survey of AIMS users, proportions and χ^2^ test results

	All	Fixed	Mobile	χ^2^	*P* a
*Who came with you to this service today?*					
Ambulance crew	0.24	0.30	0.10	9.55	< 0.01
Police	0.19	0.23	0.10	4.33	< 0.05
Street pastors/angels	0.15	0.20	0.04	8.75	< 0.01
Volunteers	0.17	0.13	0.25	5.01	< 0.05
Friends/family	0.32	0.23	0.51	16.27	< 0.01
Other	0.05	0.06	0.03	0.86	0.35
*n*	200	133	67		
*What are the reasons for being at this service today?*					
I have an injury	0.42	0.39	0.47	1.26	0.26
I feel unwell	0.13	0.10	0.19	2.92	0.09
I have been drinking alcohol	0.57	0.56	0.59	0.15	0.70
Other	0.10	0.12	0.06	1.86	0.17
*n*	202	134	68		
*Would have done if this service had not been available?*					
Looked after the problem myself	0.26	0.25	0.29	0.49	0.48
Called for help from family/friends	0.17	0.16	0.19	0.35	0.55
I would have been unsafe	0.31	0.29	0.35	0.74	0.39
Gone to hospital emergency department	0.24	0.31	0.12	8.87	< 0.01
Called the emergency services	0.15	0.15	0.15	< 0.01	0.95
Other	0.00	0.07	0.04	0.46	0.50
*n*	200	132	68		
*Who looked after you during your visit?*					
Ambulance crew	0.39	0.33	0.51	6.58	< 0.05
Nurse	0.36	0.52	0.04	44.71	< 0.001
Medical doctor	0.03	0.04	0.01	1.22	0.27
Volunteer	0.34	0.20	0.63	38.09	< 0.001
Police	0.13	0.16	0.07	2.78	0.10
Other	0.16	0.23	0.03	13.46	< 0.001
*n*	202	134	68		
*What treatment, tests or advice did you receive here today?*					
Water to drink	0.54	0.56	0.51	0.49	0.48
Referred to emergency department or hospital	0.13	0.13	0.14	0.04	0.85
Injury care (e.g. bandage, plasters)	0.24	0.21	0.31	2.16	0.14
Medication	0.07	0.08	0.03	1.95	0.16
General support	0.57	0.52	0.66	3.42	0.06
Fluids via a drip	0.03	0.04	0.02	0.74	0.39
Breathalysed or urine tested	0.03	0.04	0.00	2.53	0.11
Advice around safer alcohol use	0.17	0.17	0.17	< 0.01	0.96
Information about alcohol support services	0.06	0.06	0.06	< 0.01	0.96
Other	0.12	0.11	0.12	< 0.01	0.98
*n*	197	132	65		
*Would you have preferred to go to …*					
the local emergency department/hospital?	0.08	0.09	0.06	0.47	0.49
a different health or treatment service?	0.01	0.01	0.00	0.54	0.46
home?	0.14	0.15	0.12	0.21	0.64
I was happy to be treated here	0.78	0.75	0.82	0.91	0.34
*n*	187	122	65		
*Do you think a service like this is a good idea?*					
Yes	0.99	0.99	1.00	0.54	0.46
*n*	185	120	65		

a The threshold for significance is *P* < 0.001 following Bonferroni adjustment.

For most, the care received in the AIMS was low intensity (Table 2). For example, more than half the survey respondents indicated that they were provided with general support (e.g. having somewhere to sit, being given a sick bowl) or given water, support that suggests conveyance to ED would not be warranted. More medically oriented treatment was less common. Alcohol‐specific interventions were not routinely received, only 17% of the survey respondents recalled receiving advice on the safer use of alcohol and 6% recalled receiving information on alcohol support services.

Although care received in the AIMS was regarded positively, a minority of survey respondents would have preferred an alternative pathway (home or the ED, Table 2). Many of the survey respondents further indicated that without an AIMS, they would have sought help elsewhere, such as the ED, other emergency services or family and friends, while a quarter indicated they would have looked after the problem themselves. Almost a third indicated that they would have been unsafe if the AIMS were not available.

## Discussion

The acceptability of a new service to prospective patients is an important implementation outcome. Interview and survey data found that AIMS were acceptable to the majority of those attending. Not only did survey respondents rate their overall experience positively, most also gave eight individual aspects of care the highest possible rating and agreed AIMS were an acceptable alternative pathway to ED. Innovations in any health‐care setting require that services are acceptable to those who use them 15 and this is therefore the first study to demonstrate that alternative pathways to ED for AAI are acceptable to users.

It is notable that interview and survey data suggest that many AIMS patients neither required ED care nor would have attended ED had there been no AIMS. While AIMS may attract previously unmet need (e.g. vulnerability to assault), future research should consider the nature and extent of those needs and whether providing a location for those who are vulnerable but not requiring ED impacts on these other outcomes.

The acceptability of AIMS to users is significant particularly as interview data suggests the decision to attend an AIMS was frequently made by people other than the patient themselves. Facilitating patient choice 27 is an aspiration of health‐care systems and so identifying only a minority of survey respondents who would have preferred either a different care pathway, such as going to the ED, is notable. The decisions made on their behalf were appropriate and contributes to research indicating diverting selected AAI cases away from ED is safe 12. Some interviewees saw their care in an AIMS as a suitable intermediate response and even preferable to ED, in their view attending ED would be an unnecessary use of health‐care resources. One feature of these positive views was the interactions between patients and staff, which appeared to engender a sense of safety despite an otherwise uncertain recall of events. However, relatively few users received an intervention for their use of alcohol, and this may be associated with being unreceptive due to their level of intoxication.

There are two key strengths of the study. First, while a number of studies have recruited patrons of the NTE 28, 29, 30, this is the first to explore participant experiences as users of health services and therefore provides a novel insight into the circumstances that led patrons of the NTE into health care. Second, recruiting participants who are intoxicated presents significant practical and ethical challenges 31. Many of those attending AIMS had diminished capacity to consent at the time of referral and the window of opportunity for recruitment between attaining sobriety to consent and leaving the AIMS was quite narrow. A limitation of our findings is the potential for selection bias in reported results: those who were unwilling or unable to participate may have had fewer positive experiences of AIMS than those who participated. Others may have been referred to ED immediately following their initial assessment in the AIMS. Moreover, more men than women participated in the interviews, which might bias results towards experiences that are more likely to be experienced by male respondents. This limited opportunity to recruit and interview patients precluded opportunities to report on data saturation in qualitative interviews. However, given the challenges inherent in recruiting people to take part in such a study some degree of pragmatism is required, and we argue that the sample described here is sufficient to justify the reported conclusions. This is supported by our observation that the responses collected were consistent across survey and interview data, suggesting that respondents gave deliberate, rather than random, responses.

## Conclusion

Our study indicates that Alcohol Intoxication Management Services are acceptable to their users with many satisfied with the care and treatment received. They are also likely to capture previously unmet demand for a place of safety in the night‐time environment in addition to their stated purpose of diverting the intoxicated away from the emergency department.

## Conflict of Interest

The authors have no conflicts of interest.

## Supporting information


**Appendix S1.** Supporting Information
