# Supplementary material for: The acceptability of alcohol intoxication management services to users: A mixed methods study
Source: Drug Alcohol Rev. 2019 Nov 26;39(1):36–43. doi: 10.1111/dar.13002 (PMC7004195; doi:10.1111/dar.13002)
Supplement: Supplementary file 1 — Appendix S1. Supporting Information [file DAR-39-36-s001.pdf]

APPENDIX A

Study locations

In order to safeguard anonymity, study locations are described as Sites, where a site refers to the location of the Alcohol Intoxication Management Service (AIMS) and associated services such as emergency department (ED) and ambulance service. Six sites participated in the current study, but eight were recruited into the overall evaluation. AIMS were typically open on Friday and Saturday nights (except Site B, which was open on Wednesday and Saturday) and from late evening (8 pm Site A and Site E; 9.30 pm Site H; 10 pm Site B, Site F and Site G, 11 pm Site C) until early morning (3.30 am Site H, 4 am Site A, Site E, Site F and Site G; 6 am Site B). Two sites, D and E, are not included. They were unable to participate but in order that the current exposition is consistent with other outputs the overall study nomenclature is retained.

All AIMS had staff able to administer first aid with many including advanced nurse and paramedic practitioners. The services provided met a range of needs associated with AAI. For example, someone may become separated from friends and need no more than to charge their phone to find their way home. There were notable differences, however, in that Site A, Site F and Site B had facilities to test blood glucose levels and therefore rule out hypoglycaemia, some symptoms of which overlap with intoxication and include trembling, confusion and fatigue. Site A offered endotracheal intubation, used to maintain an open airway. As alcohol is a depressant it can promote irregular breathing, an attenuated gag reflex, can lead to aspiration of the vomitus into the lungs, and therefore asphyxiation. Intravenous saline cannot be prescribed for AAI but can be used in the event of an accelerated heart rate, and was available in site A. No AIMS provided pharmacological treatments, such as high-dose thiamine hydrochloride, benzodiazepines or naltrexone.

Site A

The AIMS opened in September 2012 as a response to the impact of acutely alcohol intoxicated patients in the local ED. It is in the centre of the city, 3.5 miles south of the ED and close to an area characterised by many licensed premises and major sporting venues. The space consists of a small seated area, six stretchers in a central treatment area with access to a single occupancy treatment room, toilets and an additional waiting area. The small seated area is used as a preliminary triage area. Once triaged, patients may be admitted and seated or laid on a bed for observation and treatment, with a preference to keep patients seated to reduce airway risks. The space can be partitioned with screens for privacy and there is seating for friends and family around the periphery of the room. There is a staff room and kitchen which functions as a hub for clinical staff stationed at the AIMS, and other staff and volunteers working in the night-time environment (NTE), e.g. local street pastors and visiting police officers. The AIMS has capacity for up to seven patients supine and up to 18 seated. The AIMS is open from 8 pm to 6 am on Friday and Saturday nights and is also open on other nights when it is anticipated the NTE will be busy. The AIMS is staffed by an Emergency Nurse Practitioner, one Health Care Support Worker and a Senior Nurse Practitioner. One police officer is stationed at the AIMS during opening hours. The ambulance service has two double crewed ambulances (four staff) stationed at the AIMS that respond to calls within the city centre.

Site B

The AIMS was established by the Police and Crime Commissioner's Office in response to the demand placed on ED, police and ambulance services due to alcohol-related assaults and AAI in the night-time environment. The intention was to divert intoxicated people away from the care of police officers and reduce the burden on ED, while also contributing to efforts to make the NTE safer. It is in the city centre, 6 miles south of the ED in an area characterised by a high density of premises licensed for the sale and on-site consumption of alcohol. The AIMS is housed in static cabins, includes toilets and a separate area designated for seating, staff and clinical activity. It is supported by a third sector ambulance service: an operations vehicle and a rapid response vehicle. Patients may wait in the seating area until triaged. Along the cabin are two clinical treatment rooms with seating and stretcher beds where patients may receive treatment and recover. There is a small staff room with kitchen facilities. The ambulance parked outside serves as an operations centre receiving 999 calls to the Site B ambulance service. Calls to the ambulance service originating from within the city centre are all redirected to the AIMS, where a response is coordinated. Police officers also use the AIMS to take witness statements, when it is appropriate to do so. The AIMS has capacity for up to three patients supine and a further five patients seated. Staff can provide treatment and support for minor injuries and support those who are vulnerable. Patients are discharged once it is safe to do so and contact has been made with friends or family members to ensure a safe transit home. The AIMS is open on Wednesdays and Saturdays, between 10 pm and 6 am. They also occasionally operate additional periods when significant events are expected to bring additional numbers into the city (e.g. sporting events and bank holidays).

Site C

The AIMS opened in November 2014 in a static ambulance. In July 2015 the service moved to premises that could also provide space for patients to recover. It operates from a building in the centre of the city 2.5 miles north of the main hospital and near to an area characterised by a number of restaurants and licenced premises. New patients are registered and triaged at the front desk. An open plan area provides seating for patients, friends and family and functions as treatment area. Mats and temporary room dividers can be used to partition areas allowing patients an opportunity to lie down. Two treatment beds are in separate rooms. The AIMS has capacity for approximately 10 patients supine and 10 seated. It is intended as an alternative pathway for those who have become vulnerable or injured, including those who are exhibiting AAI, in the night-time environment, but not as an alternative to specialist care in the ED. Street Marshalls collect and escort users into the service by foot or wheelchair. The service provides a space where patients can be monitored until they recover, given treatment for minor injuries and where arrangements can be made for a safe journey home. Typically, patients would be seated and offered water and a sick bowl. The AIMS is usually staffed by up to three substance misuse workers, one nurse, two medics and security staff.

Site F

The AIMS was established in 2010 by the local police and ambulance services to relieve pressure on the ambulance service from AAI on Friday and Saturday nights and as a place of safety. The AIMS is a collection of vehicles: a large mobile ambulance treatment unit, an additional ambulance and a police van. The AIMS (19 May 2017 onwards) is situated in the city centre, a mile south east of the ED. A third sector organisation provides a treatment unit used for initial triage, seating for minor injury treatment, a waiting area and two stretchers for further treatment and recovery. The ambulance typically receives calls via the ambulance service and city linked radio to attend and bring intoxicated or injured patients back to the AIMS or direct conveyance to ED. The police van provides a space where vulnerable patients can wait in a safe environment. Those with uncomplicated acute intoxication can sit and recover before safe transport home can arranged. The AIMS has capacity for approximately three patients supine and eight seated. Furthermore, the AIMS provides a service in which people can seek assistance if they are feeling vulnerable or they are lost. In addition to medical assistance people also can speak to a police officer in confidence. Typically, patients may receive treatment for minor wounds and then be offered a place to sit and recover in the police van. Typically, the AIMS would be staffed by two to five paid staff, one ambulance Emergency Medical Technician (who assumes a clinical lead role and takes responsibility for the facility), an Emergency Transport Assistant and up to four other volunteer first aiders (advanced and non-advanced). The police van is run by two police officers.

Site G

The AIMS was opened in December 2014 as an extension of an existing Street Pastor scheme and in response to the perceived burden that AAI patients from the NTE was placing on ED. In addition, local Street Pastors raised concerns that most of their time was taken waiting for ambulances and trying to meet the needs of ill and injured patrons on the street, which is not seen as their core purpose. The AIMS was designed to help vulnerable people in the night-time environment, as well as reduce the impact on emergency services of those who have consumed excessive amounts of alcohol or have used illicit substances. Unlike the other AIMS, the local ambulance service did not refer patients into the AIMS. It is in a building in the town centre, half a mile west of the ED and close to licensed premises and fast food outlets. The space consists of a main reception area with seating around the sides. A partitioned area contains the office equipment, CCTV and a viewing window into an adjoining recovery room. The recovery room contains three crash mats on the floor. There is a small single occupancy treatment room with a stretcher bed, medical supplies and a further small room. There is a kitchen and toilet for staff and patient use. A large people carrier style vehicle is stationed outside. The main reception area is used to initially seat and assess patients in which waiting friends and relatives can wait. After initial triage patients may be taken to the recovery room and laid on crash mats or taken to the treatment room for first aid. The additional room may be used for people who may require privacy and emotional support. Staff, patients, friends and visiting Street Pastors use the main reception area to congregate and await referrals. The vehicle is used by staff to pick up patients from the city centre and as a means to transport patients home. The AIMS has capacity for approximately five patients supine and a further five seated. It is open every Saturday, some Fridays, bank holidays and Sunday nights from 10 pm to 4 am. The service is staffed by between four and 10 volunteers including a senior shift leader and a team of local college students and has managerial and administrative support from the lead organisation. There is no clinical input.

Site H

The service was established following the death of three young adults in late 2000. Two men drowned in the river during a night out drinking, and a third youth was found dead in a city centre nightclub due to

alcohol intoxication. The AIMS bus is intended to keep people safe and reduce unnecessary ambulance callouts by assisting anyone at risk. It is a collection of three buses: the bus, the medical unit and a mobile support vehicle (minibus van). All three vehicles park in a layby in the city centre, 5 miles west of the ED. The bus provides a long narrow space with bench seating to triage and monitor patients. The medical unit contains two stretcher beds, used to administer treatment and provides additional space for recovery. The mobile support van usually responds to calls from licenced premises and local street-based volunteers for assistance and returns patients to the bus for assessment. The bus has capacity for two supine and three seated patients but will use public seating outside the bus with appropriate staff support as an additional waiting area. The bus team consists of first aid volunteers and community first responders, who are supported by an ambulance paramedic and a private security guard. A volunteer shift leader from a charity is supported by other shift support volunteers including a driver who can operate the mobile unit.

APPENDIX B

AIMS user survey

The University Of Sheffield.

CARDIFF UNIVERSITY PRIFYSGOL CAERDYDD

Please tell us your views about [service name]

1. Who came with you to this service today? *(Please tick all that apply)*

Ambulance crew		Volunteers	
Police		Friends/family	
Street pastors/angels		Other (Please describe) ____________	

2. What are the reasons for being at this service today? *(Please tick all that apply)*

I have an injury (e.g. sprained ankle)		I have been drinking alcohol	
I feel unwell		Other(Please describe) ____________	
		____________	

3. What do you think you would have done if this service had not been available? *(please tick all that apply)*

Looked after the problem myself		Gone to hospital emergency department	
Called for help from family/friend/other		Called the emergency services e.g.999	
I would have been unsafe		Other(Please describe) ____________	

4. Who looked after you during your visit? *(Please tick all that apply)*

Ambulance crew		Volunteer	
Nurse		Police	
Medical Doctor		Other(Please describe)____________	

5. What treatment, tests or advice did you receive here today? (please tick as many as apply)

Water to drink		Fluids via a drip	
Referred to emergency department or hospital		Breathalysed or urine tested	

6. How would you rate the service on each of the following? (Please tick a box on each line)

		Very good	Fairly good	Neither good nor poor	Fairly poor	Very poor
A	Service location					
B	Safety					
C	Comfort and cleanliness					
D	Communication (e.g. being told what is happening)					
E	The care and compassion of the staff who looked after you					
F	The tests and treatments received					
G	Any advice or information provided					
H	How you were discharged/ when you left					

7. Would you have preferred to go to… *(please tick one)*

The local emergency department/ hospital	☐	Home	☐
A different health or treatment service	☐	I was happy to be treated here	☐

8. In general do you think a service like this is a good idea? **Yes** ☐ **No** ☐

9. ***Overall … (please circle a number)***

I had a very poor experience — I had a very good experience

0	1	2	3	4	5	6	7	8	9	10

10. What do you think was good about the service?

What could be improved about the service?

Any other comments:

ABOUT YOU:

11. How old are you? …………years

12. Are you Male ☐ Female ☐

Please return the completed questionnaire inside the envelope in the box provided, or complete and post in the **FREEPOST** reply paid envelope attached.

Thank you for your help
